# Detection of impurities in dietary supplements containing l-tryptophan

**DOI:** 10.1007/s00726-022-03125-9

**Published:** 2022-01-18

**Authors:** Sachise Karakawa, Akira Nakayama, Naoto Ohtsuka, Katsuma Sato, Miro Smriga

**Affiliations:** 1 International Council on Amino Acid Science (ICAAS), Avenue de Tervueren, 188A, 1150 Brussels, Belgium; 2 Research Institute for Bioscience Products & Fine Chemicals, Ajinomoto Co., Inc., 1-1 Suzuki-cho, Kawasaki-ku, Kawasaki-shi, Kanagawa, 210-8681 Japan; 3 Shimadzu Techno-Research Inc., 3-19-2 Minamirokugo, Ohta-ku, Tokyo, 144-0045 Japan

**Keywords:** Tryptophan, Dietary supplements, Impurity analysis, HPLC

## Abstract

Impurities in nine dietary supplements containing l-tryptophan were evaluated using an HPLC methodology. In five tested products, the total impurities were higher than the thresholds described in the Food Chemical Codex or implemented in the EU for pharmaceutical grade l-tryptophan. In addition, liquid chromatography–mass spectrometry was used to specifically test for the presence of 1,1′-ethylidenebis-l-tryptophan (EBT). None of the tested products contained detectable amounts of EBT. High amounts of unidentified impurities in some dietary supplements point to potential health risks.

## Introduction

The not-for-profit association “International Council for Amino Acid Science” (ICAAS) promotes research on the safe use of amino acids in human nutrition (e.g., Deutz et al. 2017; McNeal et al. 2018; Gheller et al. 2020). In addition, ICAAS contributed to an assessment of purity standards of amino acids (Smriga 2020). The goal of this study was to evaluate the purity of nine globally available dietary supplement brands containing l-tryptophan (Trp), which were chosen due to the presumed links between Trp impurities and cases of eosinophilia-myalgia syndrome (EMS) (Varga et al. 1992; Allen et al. 2011).

Trp is an essential amino acid (Wu 2021) ingested in regular diets at 0.9–1.0 g/day (DRI 2005). This intake satisfies the nutritional requirements of most children and adults (Al-Mokbel et al. 2019), yet many people choose to enhance their dietary intake of this essential amino acid by means of dietary supplements due to motivations that are beyond the scope of this study (Fernstrom 2012). The US National Institute of Health’s database of dietary labels (NIH 2021) revealed more than 800 different dietary supplements containing Trp at a dose equal to or below 0.5 g/day. A sub-chronic clinical safety trial (Hiratsuka et al. 2013) determined that the upper limit of safe intake of supplemental Trp in normally fed adults was as high as 5.0 g/day, and thus this study assumed that the health risk (if any) of Trp supplementation was associated with impurities rather than overdosing (Smriga 2020). In addition to evaluating total impurities, liquid chromatography–mass spectrometry (LC–MS) was used to evaluate the presence of 1,1′-ethylidenebis-l-tryptophan (EBT), a toxic impurity previously linked to EMS outbreaks (Sidransky et al. 1994).

## Materials and methods

### Chemicals, reagents and standards

Nine commercial Trp dietary supplements were purchased online (September 2020). The selected dietary supplements were manufactured in three regions (US, Europe and Japan) and sold globally. The products contained Trp at 500 or 1000 mg as a daily recommended dose (either as a tablet, a capsule or a powder sachet). Three products contained Trp alone, while others also included vitamins, minerals and/or other amino acids (specifically, l-lysine). *N*-Acetyl-dl-tryptophan (*N*-Ac-dl-Trp) and pyridoxal-5′-phosphate were purchased from Nacalai Tesque (Kyoto, Japan). 3,3′-[Ethylidenebis(1H-indole-1,3-diyl)]bis[2S]-2-aminopropanoic]acid (= EBT) standard and 2-[2,3-dihydroxy-1-(3-indolyl)-propyl-l-tryptophan (dhPIT) were synthesized in-house. EBT; ^1^H NMR, (600 MHz, D_2_O) δ 7.69 (dd, *J* = 8.0, 4.4 Hz, 2H), 7.54–7.48 (m, 2H), 7.45 (s, 2H), 7.27–7.22 (m, 2H), 7.22–7.13 (m, 3H), 4.02 (dd, *J* = 7.5, 5.4 Hz, 2H), 3.48–3.38 (m, 2H), 3.32–3.24 (m, 2H), 2.19 (d, *J* = 6.5 Hz, 3H), MS, [M + H]^+^ = 435, dhPIT; MS, [M + H]^+^ = 394. Acetonitrile (LC/MS grade), trifluoroacetic acid (TFA), formic acid (LC/MS grade), distilled water, nicotinamide, l-ascorbic acid, riboflavin, folic acid, thiamin hydrochloride, and pyridoxine hydrochloride were obtained from FUJIFILM Wako Pure Chemical Corporation (Osaka, Japan). Taurine and l-lysine were purchased from SIGMA (St. Louis, MO, USA).

The standard primary stock solution of *N*-Ac-dl-Trp (20 mg/dL) was diluted 1–20 and thereafter 1–10 to give a final standard solution (100 μg/dL, equivalent to 100 ppm in a sample). In accordance with the FCC methodology (12th edition) and to enable comparisons, we are using ppm units, instead of μΜ, throughout this manuscript.

The stock solution of EBT (1 mg/dL) was diluted 1–10 to make a standard solution (100 μg/dL, equivalent to 100 ppm in a sample). Nicotinamide, l-ascorbic acid, riboflavin, folic acid, thiamin hydrochloride, pyridoxine hydrochloride, pyridoxal-5′-phosphate, taurine, and l-lysine were individually dissolved in water and prepared at final concentrations of 10 mg/dL, equivalent to 100 ppm in a sample. Finally, each test sample was weighed as 1.0 g of Trp. Water was added up to 100 mL and filtered through a 0.20 μm filter to prepare a sample solution (10,000 ppm solution).

### HPLC analysis

The analytical method corresponded to the US Pharmacopoeia (USP24) and the FCC. A Prominence HPLC system from Shimadzu Co., Inc. (Kyoto, Japan) was used. Mobile phase A consisted of 0.1% TFA in water and mobile phase B consisted of 0.1% TFA in acetonitrile/water (80/20, v/v). The analytical column (Ultrasphere, 5 μm, 250 mm × 4.6 mm i.d.; Hichrom, Berkshire, UK) was used at 30 °C. The flow rate was 1.0 mL/min. The gradient conditions were as follows: 0–2 min maintained at 5% mobile phase B, 2–37 min from 5 to 65%, 37–42 min from 65 to 100%, 42–47 min at 100%, 47–50 min from 100 to 5%, and 50–60 min at 5% for re-equilibration. The injection volume was 20 μL. The UV detection wavelengths were 220 nm and 280 nm. LC Solution (Shimadzu) and Microsoft Excel were used for data processing.

### Liquid chromatography–mass spectrometry (LC–MS) analysis

A Nexera X2 HPLC system from Shimadzu was connected to a Sciex Triple Quad 6500 MS/MS system (Sciex, Framingham, MA, USA). Analyst 1.6.2 software (Sciex) was used to control these instruments. The analytical column (YMC Triart PFP column, 1.9 μm, 100 mm × 2.1 mm i.d.; YMC, Kyoto, Japan) was used at 40 °C. The flow rate was 0.25 mL/min. Mobile phase A consisted of 0.35% formic acid in water and mobile phase B consisted of acetonitrile/water (95/5, v/v). The gradient conditions were as follows: 0–3 min maintained at 0% mobile phase B, 3–8 min from 0 to 50%, 8–9 min from 50 to 95%, 9–10 min at 95%, 10–10.1 min from 95 to 0%, and 10.1–15 min at 0% for re-equilibration. The injection volume was 3 μL. The mass spectrometry parameters were as follows: curtain gas was 40, ion spray voltage was 5500 V, temperature was 600 °C, ion source gas 1 was 50, ion source gas 2 was 70, and collision-activated dissociation was 7. Trp impurities and their metabolites were qualitatively analyzed using MS scans (scan range from *m*/*z* 100 to 500) and MS/MS in positive mode.

## Results

### Total impurities

**Fig. 1:**
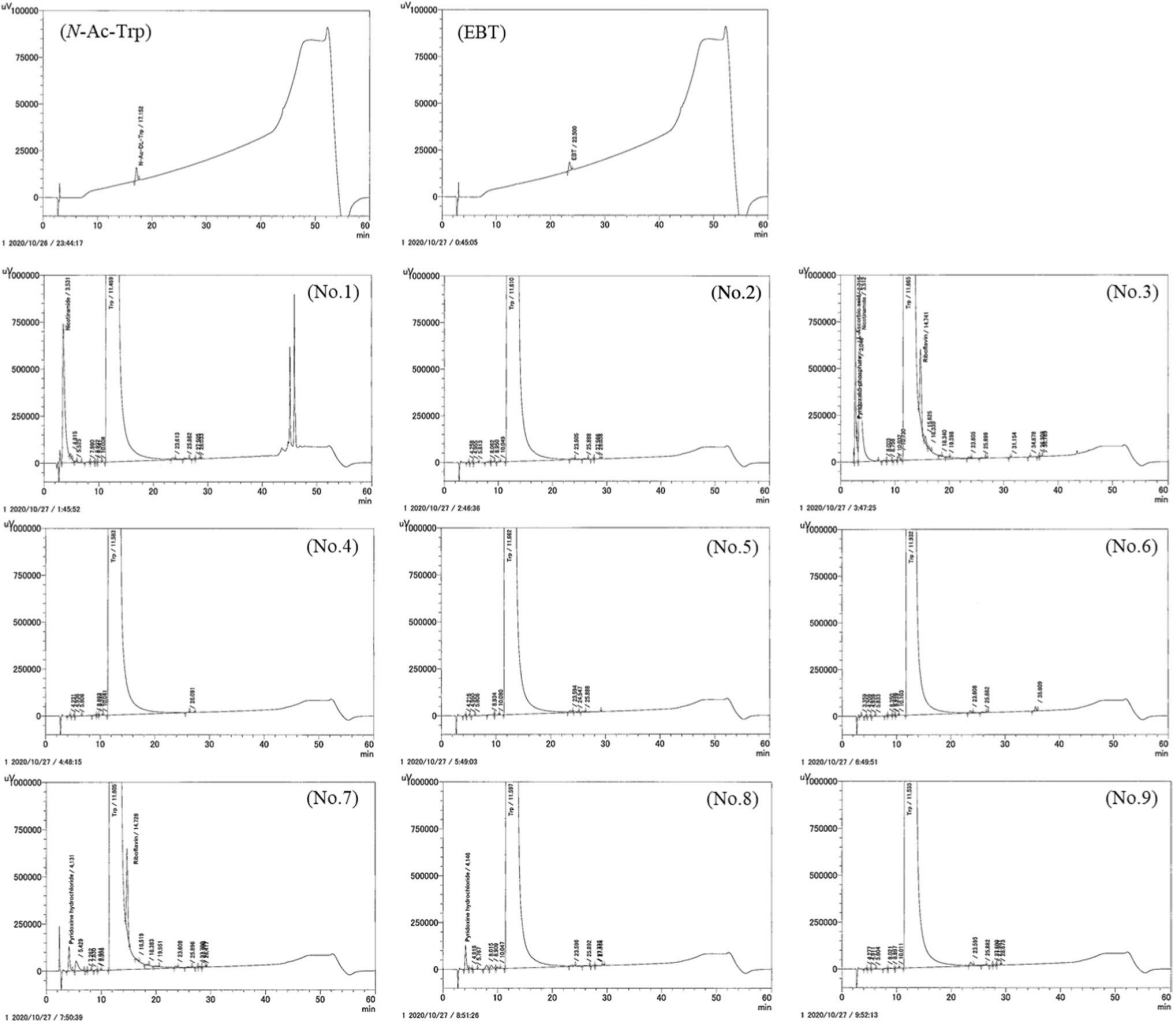
UV (220 nm) chromatograms of *N*-Ac-Trp (100 ppm) and EBT (100 ppm) standard solutions and the nine Trp samples. The impurity peaks detected from 0 to 11 min were integrated and designed as the “before Trp peak” area. The peaks detected after Trp (from 14 to 40 min) were integrated as the “after Trp peak” area

Chromatographs of the sample solutions are shown in Fig. 1. Sample solution No. 1 contained nicotinamide, sample No. 3 contained ascorbic acid, pyridoxal-5′-phosphate, nicotinamide and riboflavin, sample No. 7 contained pyridoxine hydrochloride and riboflavin, and sample No. 8 contained pyridoxine hydrochloride which were listed on their label and the corresponding peaks were excluded from the quantitation. When a component not considered to be an impurity (due to its high signal intensity) was detected, the value at UV 280 nm was confirmed. In addition, if the signal intensity ratio (UV 220 nm/UV 280 nm) differed markedly from that of *N*-Ac-Trp (UV 220 nm/UV 280 nm = 5.56) and was outside the range of 1.5–10, this component was also excluded. The total amount of impurities (converted to *N*-Ac-Trp) before the Trp peak in each product was 13.9–931.4 ppm, and the total amount of impurities after the Trp peak was 7.3–846.7 ppm (Table 1).

**Table 1:** Total impurities and EBT peak in the tested Trp products

No	Total impurities (ppm)		EBT (ppm)
	Before Trp peak	After Trp peak	
1	182.5	199.1	–
2	97.6	123	–
3	517.7	846.7	–
4	73.5	7.3	–
5	18.5	182.5	–
6	13.9	202.7	–
7	80.5	359.7	–
8	931.4	161.6	–
9	88.4	317.1	–

–, not detected

### Identification of peaks near EBT

A peak with a retention time similar to that of EBT was observed using the described HPLC methodology and thus the presence or absence of EBT in these samples was confirmed using LC–MS analysis. However, it was difficult to detect impurities at UV 220 nm due to interference by absorption by formic acid in the mobile phase and thus UV 280 nm was used instead. An EBT peak was identified at 8.7 min using the EBT standard solution, but no such peak was found in any of the Trp commercial products (data not shown). On the other hand, an impurity peak with a retention time similar to that of EBT was detected at 8.9 min in eight of the nine Trp samples. The *m*/*z* of this impurity peak was 394, which was different from that of EBT (*m*/*z* 435). An impurity with a molecular weight of 393 was previously reported to be dhPIT (Simat et al. 2003). The peak was identified using dhPIT standards and the retention time, mass spectrum and MS/MS spectrum matched (data not shown). The dhPIT peak was detected in all samples except for Trp product No. 4.

## Discussion

The aim of this study was to build upon the peer-reviewed literature (Rogers and Smriga 2012) by evaluating the total impurities in commercially available Trp supplements, which were chosen to represent dietary supplement products manufactured in the US, EU and Japan and available by e-commerce globally. The study utilized an HPLC methodology described in the FCC monograph (12th edition) and assessed the presence of EBT using LC–MS. Although the presence of EBT was not detected, five of the nine evaluated brands were characterized by total impurities at amounts higher than the threshold levels specified in the 12th edition of the FCC monograph, which is important because the causative link between a specific impurity and the onset of EMS has not been fully established (Fernstrom 2012). As a precautionary measure, the FCC and European regulation limit the total HPLC UV-detectable impurities to certain maximum amounts eluting before and after the Trp peak.

Although the evaluated Trp-containing products represent a small segment of commercial products (NIH 2021) the results point towards a need to improve the enforcement of purity standards for Trp and other functional ingredients in dietary supplements (Rogers and Smriga 2012). Alas, many national authorities regulate ingredients in dietary supplements only by establishing maximum limits on daily intake (Smriga 2020). Using Trp as a typical ingredient in supplements, this is a paradoxical regulatory approach since a controlled clinical safety study in healthy adults described a substantial safety margin for high-purity supplementary Trp equal to more than 5-times its intake from regular food sources (Hiratsuka et al. 2013). The same applies to numerous other proteinogenic amino acids (e.g., Gheller et al. 2020; Miura et al. 2021).

In summary, we found high amounts of total impurities in more than half of the tested Trp-containing dietary supplements. The results call for more stringent and better coordinated international control of the quality and purity of nutrients used in dietary supplements.

## Data Availability

Available upon request.
